# Decompression, an Unusual Treatment Option for Multicystic Ameloblastoma: Concepts and Controversies

**DOI:** 10.1155/2024/7126223

**Published:** 2024-10-12

**Authors:** Cinthya María Quisiguiña-Salem, Alejandro Alonso-Moctezuma, Carla Monserrat Martínez, Fabiola Salgado-Chavarría, Itzel Legorreta-Villegas, James Jerez-Robalino

**Affiliations:** ^1^Department of Oral and Maxillofacial Surgery, Postgraduate Studies and Research Division (DEPeI), Faculty of Dentistry, Universidad Nacional Autónoma de México, Mexico City, Mexico; ^2^Department of Pathology and Oral and Maxillofacial Medicine, Postgraduate Studies and Research Division (DEPeI), Faculty of Dentistry, Universidad Nacional Autónoma de México, Mexico City, Mexico

**Keywords:** ameloblastoma, case report, conservative treatment, decompression, quality of life

## Abstract

**Background:** The most common odontogenic tumor is the solid ameloblastoma. This is concerning due to the progressive bone destruction in its intraosseous variant; this type of pathology often gets a delayed diagnosis due to the asymptomatic characteristic it has.

**Objective:** The aim of the article is to propose a conservative treatment for multicystic ameloblastoma, reviewing the latest concepts, controversies, and treatment options described in the literature.

**Case Report:** A patient arrives to our department with a diagnosis of unicystic ameloblastoma, so decompression and subsequent enucleation were decided as treatment. However, the new histopathological result was a multicystic ameloblastoma; with this result, we decided to continue with the initial treatment, which in fact had an adequate response. A 5-month radiographic follow-up was performed in which a decrease in size was evident and enucleation was decided. The postoperative results were favorable after a 2.5-year follow-up.

**Conclusion:** The solid ameloblastoma may have a cystic component, in which there is the possibility of diagnostic errors when studying an incisional biopsy; for this reason, the definitive diagnosis should be established until complete enucleation of the lesion is performed. This characteristic in a solid ameloblastoma makes possible a positive response to decompression, making it easier to perform the enucleation subsequently, avoiding other aggressive treatments that can dramatically affect the patient's quality of life and also avoiding costly reconstruction bills.

**Summary**

• Treatment selection should be made considering the biological, socioeconomic, and psychological factors of the patients.
• Decompression as a treatment for multicystic ameloblastoma has shown favorable results, and it also has low morbidity and cost; therefore, it should be considered a treatment option.
• Radical treatment could be chosen until a therapeutic decompression failure is determined.
• It should be considered that the tumor is heterogeneous and there may be variability in the results between the incisional and final biopsy after its definitive treatment.
• Ameloblastomas require long-term follow-ups due to their high recurrence rate.

## 1. Introduction

In 1937, Robinson described ameloblastoma as a benign tumor “usually unicentric, nonfunctional, intermittent in growth, anatomically benign, and clinically persistent” [1]. It is one of the odontogenic tumors of epithelial origin that derives from cells that form dental organs without a mesenchymal component [2]; it can originate from the enamel organ, the Malassez remains, Hertwig's epithelial sheath, and the basal cells of the oral mucosa or the epithelium of odontogenic cysts [3].

Ameloblastoma generally appears in male patients, with a great predilection for the posterior region of the jaw. It can also appear in the maxilla and less frequently in other sites such as the middle ear [4] and paranasal sinuses [5, 6]; its localization will be altered by genetic factors and the syndromes involved [7, 8].

It has a slow growth pattern and can generate cortical bone expansion, causing facial deformity. The initial diagnosis is generally made by X-ray imaging, seen in an orthopantomography or a computed tomography (CT), confirmed only by its histopathology [2]. This tumor management is widely discussed; there is a radical treatment described, in which enucleation of the lesion is performed leaving up to 3-cm free margins; this is to reduce the possibility of recurrence due to the presence of small tumor nests beyond its edges.

In 2022, the World Health Organization (WHO) modified the ameloblastoma classification, adding adenoid ameloblastoma as a separate entity, thus classifying the ameloblastoma as conventional, unicystic, peripheral, adenoid, and metastatic [9].

The unicystic ameloblastoma has an infiltration of approximately 0.25 cm in bone, without exceeding 0.5 cm; meanwhile, in multicystic or solid variants, it will depend on the histological subtype, the plexiform or follicular reporting 0.5-cm infiltration and the granular with a greater infiltration rate of up to 0.75 cm, which is why a marginal resection of 1–2 cm is recommended. However, each case must have a personalized treatment, depending on the histology and patterns it may present [10]; it is necessary to mention that when a solid ameloblastoma has a cystic component, it could respond positively to a decompression avoiding a mutilating or aggressive treatment.

For this reason, we attribute the resolution of the tumor in the case presented in this publication, agreeing with previous results carried out by Huang et al., who opted for a conservative therapy with decompression and enucleation of the multicystic ameloblastomas obtaining similar results to ours but in a pediatric population [11, 12]; however, recurrence is questioned by some authors. Others, on the contrary, opt in the first instance for conservative treatment, especially in the pediatric population [2, 11–15].

The objective of this case report is to propose conservative treatment for solid ameloblastoma with a cystic pattern through decompression followed by its enucleation. In addition, the most relevant and new concepts of ameloblastoma will be analyzed, in terms of its classification, characteristics, diagnosis, genetics, and treatment options.

## 2. Case Report

**Figure 1:**
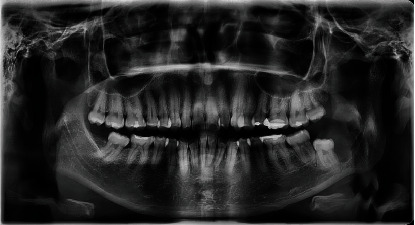
Orthopantomography image before Tooth #38 was extracted. Note the multicystic radiolucency on the left mandibular ramus.

**Figure 2:**
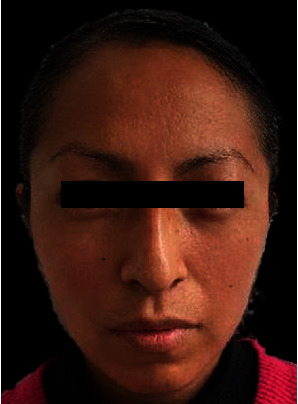
Frontal facial photography with no evident facial deformation.

**Figure 3:**
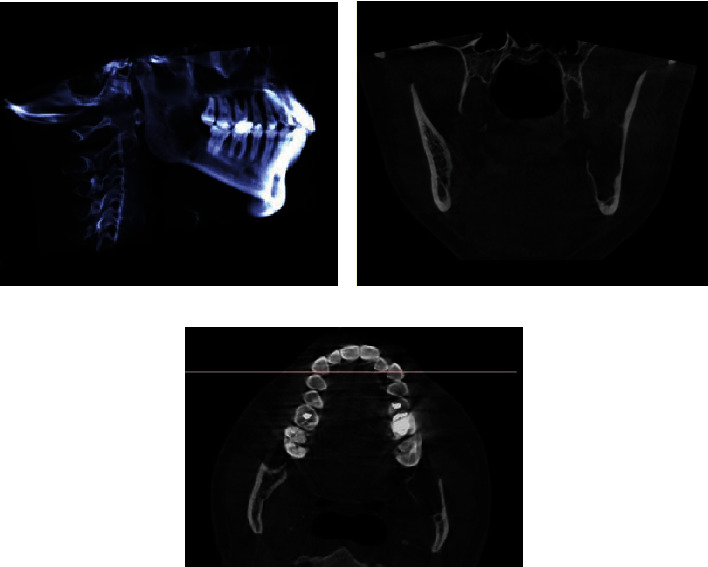
With computed tomography, the extension and involvement of the lingual cortical bone can be observed. (a) Sagittal view. (b) Coronal view. (c) Axial view.

**Figure 4:**
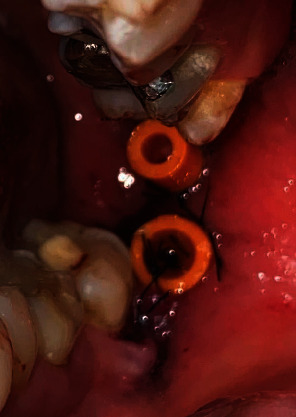
Decompression devices in position, placed in a cephalic and caudal direction.

**Figure 5:**
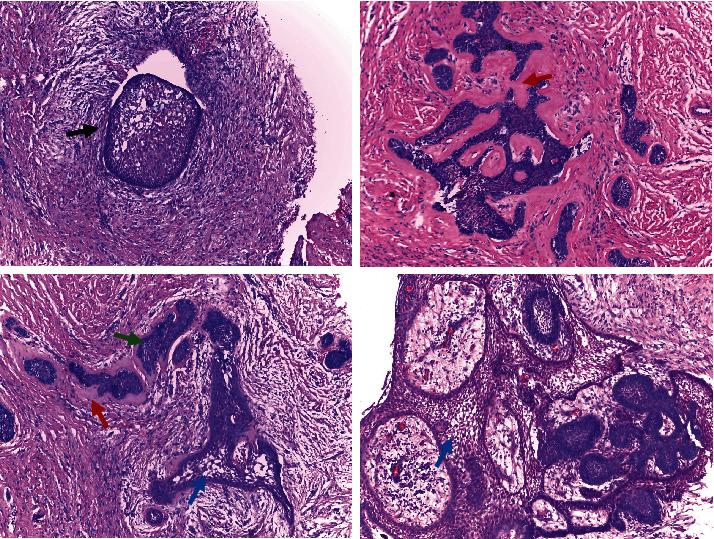
Conventional ameloblastoma. Nests (black arrow); anastomosing cords (^∗^) with columnar cells at its periphery, with palisade and hyperchromatic nuclei (green arrow); discohesive areas, stellate reticulum (blue arrows); and dense connective tissue with hyaline areas (red arrows). The cystic pattern is observed.

This case report was written following the CARE guidelines [16, 17]. A 36-year-old woman with no personal or hereditary pathological history, who attended the Cuautitlán Izcalli Cleft Lip and Palate Clinic, State of Mexico, at the Maxillofacial Surgery Service for diagnosis and treatment of a mandibular lesion, was previously diagnosed with an incisional biopsy that was taken during a 38-tooth extraction by another maxillofacial surgeon in his private office; the histopathological result showed a “unicystic ameloblastoma” (Figure 1). At the physical examination, no facial deformity was observed (Figure 2), and no neck lymphadenopathy was palpable. Intraorally, a scar was evident at the left retromolar region corresponding to the previously performed extraction. A simple CT of the face was requested, where a well-circumscribed hypodense area was observed when compared to the adjacent bone structure; it extended from the mandibular ramus in its anterior half, without reaching the sigmoid notch, showing loss of the lingual cortical continuity of the ramus (Figure 3). The informed consent was obtained, and based on the previous diagnosis, we decided to perform the decompression of the pathology under local anesthesia placing two tube-like devices for this purpose, made with a Nelaton No. 14 probe; both devices were placed in the cavity, one of which was placed in a cephalic direction and the other in a caudal direction (Figure 4); a second incisional biopsy was obtained to confirm the first diagnosis; the result was a conventional ameloblastoma with a follicular pattern (Figure 5). We decided to continue with the initial treatment, because the patient showed an adequate response to decompression.

**Figure 6:**
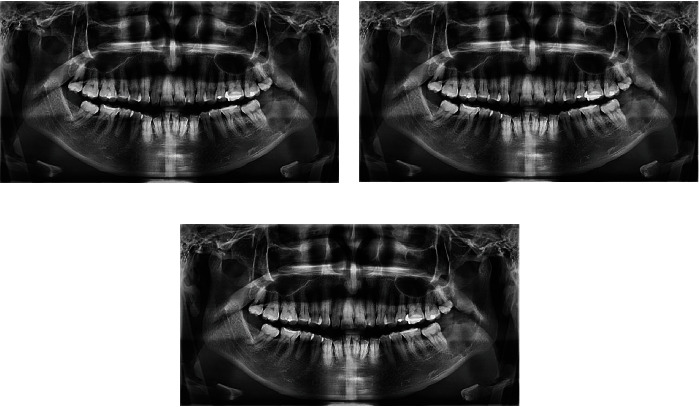
After decompression follow-up orthopantomographies. (a) 1-month follow-up. (b) 2-month follow-up. (c) 3-month follow-up.

The patient was instructed how to irrigate through the drainage tubes using 0.9% physiological solution every 8 h with a 20-mL syringe. Clinical and radiographic follow-ups were performed monthly for 5 months, until cyst reduction stopped (Figure 6).

**Figure 7:**
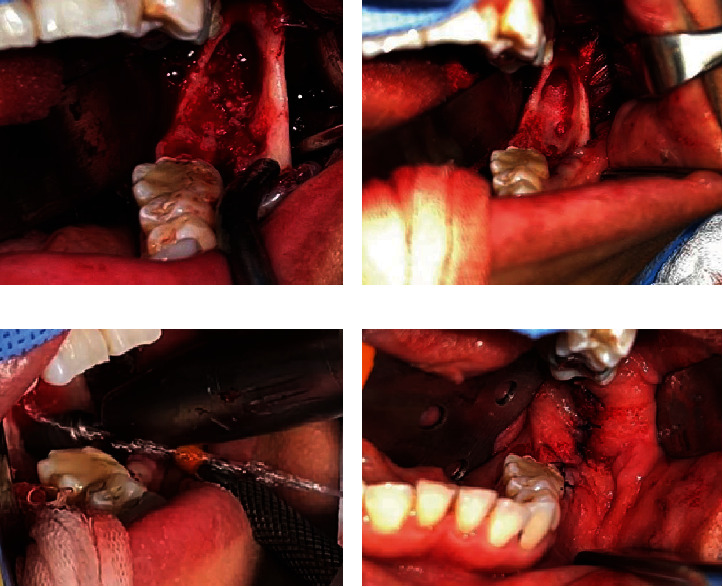
Enucleation. (a) Surgical approach. (b) Enucleation and curettage. (c) Peripheral ostectomy. (d) Closure with 4-0 Vicryl.

Subsequently, enucleation with curettage was performed under general anesthesia; during surgery, we decided to extract Tooth Number 37 because Miller's grade II mobility was seen and the tumor was in contact with it. This was done to reduce the tumor cell persistence that could lead to a recurrence. Following the enucleation, a 5-mm peripheral ostectomy was performed with a ball burr; its depth was controlled by making osteotomies with a 702 burr only in places where the patient's anatomy allowed it (Figure 7). All the biological tissues collected in this procedure were sent for histopathological study, whose report showed only fibrous tissue.

**Figure 8:**
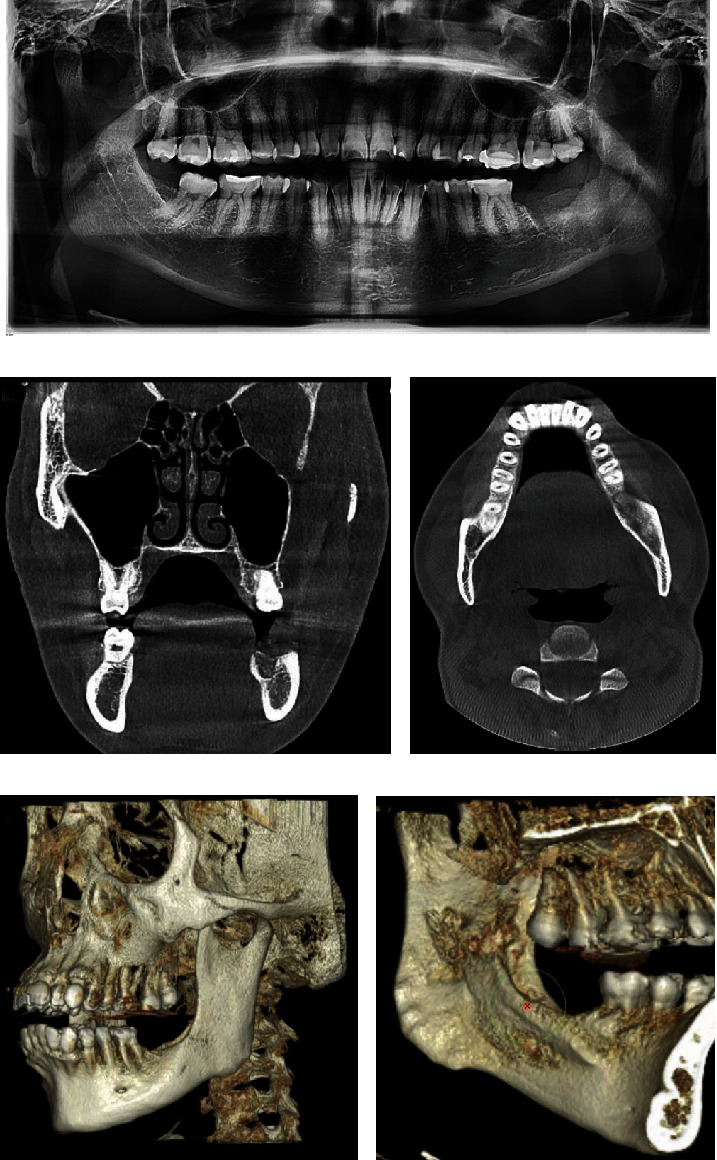
Posttreatment imaging studies. (a) 7-day postoperative panoramic radiograph demonstrating the socket of the second molar. (b, c) 4-month postoperative CT scan and coronal and axial cuts revealing increased radiodensity at the surgical site. (d, e) Isovolumetric reconstruction showing the integrity of the buccal and lingual cortical bone.

A 7-day postoperative orthopantomography was obtained, and a 4-month postop CT scan was made; this delay to obtain a CT scan was due to the institution's lack of resources and because the patient has a low socioeconomic status (Figure 8).

**Figure 9:**
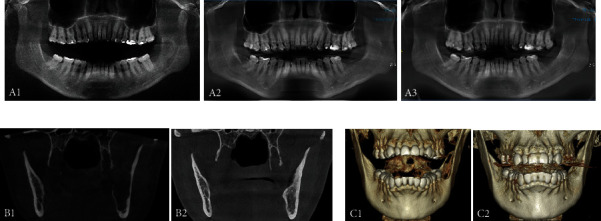
Before and after radiographic and tomographic image comparison. (a) Panoramic radiographic comparison. (A1) Before treatment. (A2) 1.5-year follow-up after treatment. (A3) 2.5-year follow-up after treatment. (b) Coronal tomographic cuts. (B1) Before treatment. (B2) 2.5-year follow-up after treatment. (c) 3D isovolumetric reconstruction. (C1) Before treatment. (C2) 2.5-year follow-up after treatment.

The patient was in good local and general condition, so discharge was indicated the next day after the surgery. A tomographic follow-up every 6 months for 2.5 years was performed, during which no signs of recurrence were detected, and bone density improvement was evident (Figure 9).

Next, the decompression/enucleation technique used by the authors is described below step-by-step:

First, all materials to be used must be prepared: a 14- or 16-gauge Nelaton or Foley catheter, a 3-0 nylon suture, and local anesthesia (depending on the treatment area). Surgical access is achieved through a sulcular incision with a posterior retromolar discharge and subperiosteal dissection, avoiding tumor perforation, especially in areas with cortical perforation.

Tumor exposition is achieved through a superficial perilesional osteotomy, avoiding tumor lacerations. In our case, an incisional biopsy was taken for diagnosis confirmation. This was obtained by making a deep spindle-shaped incision with a No. 3 scalpel handle and a No. 11 blade; this blade was used to obtain the largest possible amount of tissue for histopathological analysis. The distance of the Nelaton probe is then measured with the selected diameter; then, it is cut to a length long enough to reach the base of the tumor. Perforations are made at the end of the probe using Mayo scissors to assure the deepest contact at the base of the tumor, allowing irrigation throughout the lesion. If necessary, several drains must be placed to facilitate irrigation. Probe fixation is made with a 3-0 or 4-0 nylon suture, perforating the probe without obstructing its lumen and then suturing it to the oral mucosa. The soft tissue is closed with a 4-0 Vicryl suture. To verify the permeability of the drains, saline solution is irrigated through the placed probes. At the end, the patient is instructed to irrigate through the drains 20 mL of 0.9% saline solution every 8 h. After the procedure, antibiotics and analgesics of choice are prescribed.

For decompression monitoring, a panoramic X-ray is recommended every 2 months until the size stops decreasing. General anesthesia is recommended for enucleation and curettage. However, local anesthesia may be used for smaller lesions without anatomical risks.

Enucleation is performed with a delicate dissection, avoiding tumor tearing or perforation. The tissue obtained is then sent for histopathological study. A silicone endodontic file stop is attached 5 mm from the tip of a 702 drill, and ostectomies are then performed until contact is made with the silicone stop. Rotary curettage is performed using a ball drill, reaching a depth of 5 mm at the base of the osteotomies. In areas close to the adjacent tooth roots and near the lower dental nerve, only curettage is performed with a Lucas curette. Tooth extraction is only recommended if the tumor has not shrunk enough, and the tooth remains inside or in contact with it. Surgical access closure is made with a 4-0 Vicryl suture.

## 3. Current Concepts

### 3.1. Definition

The ameloblastoma is a slow growing, locally invasive, benign epithelial odontogenic tumor with a high recurrence potential [7, 18]. It can derive from the cellular remains of the enamel organ, the dental lamina, Hertwig's sheath, the Malassez remains, the basal cells of the oral epithelium, and the epithelium of preexisting odontogenic cysts or heterotopic epithelium of other parts of the body such as the pituitary gland [3, 7, 19].

### 3.2. Epidemiology

Ameloblastoma is the most common odontogenic tumor (excepting the odontoma) according to the WHO [9], with an incidence of 0.92 cases per 1 million people per year [18]. A male predilection is reported in Africa, North America, Asia, and Australia; however, in South America and Europe, women are found to be the most affected, but with no statistical significance. The average affected age varies around the world, being more common in the third decade of life in South America and Africa, while in Europe and North America, it occurs more at the fifth and sixth decades of life [20]. Geographically, there are incidence differences, which is why in countries such as Africa, China, and India, this tumor is more common than in the western world [21].

### 3.3. Classification and Histological Characteristics

Histologically, the ameloblastoma is characterized by having an epithelium resembling the epithelial components of the enamel organ in a fibrous stroma; the epithelial lining is composed of columnar cells with hyperchromatic palisade nuclei with inverted polarity and subnuclear vacuolation [9].

In 2022, the WHO classifies the ameloblastoma into five variants: solid/conventional/multicystic with six histological subtypes, unicystic with three subtypes, peripheral, adenoid, and metastatic. The histopathological subtypes of conventional ameloblastoma are plexiform, follicular, acanthomatous, desmoplastic, granular, and basal cells. The follicular pattern is the most common (39%) followed by the plexiform pattern [9] (Table 1).

In the fifth edition of the *WHO Classification of Head and Neck Tumours*, the adenoid ameloblastoma was added as a new recognized entity separate from solid ameloblastoma, because it does not share the BRAF p.V600E mutations found in other ameloblastomas. This pathology can be confused with an adenoid odontogenic tumor, a dentinogenic ghost cell tumor, or an odontogenic carcinoma with dentinoid, due to the histological similarities they share; however, in the adenoid ameloblastoma, a combination of characteristics must be considered, such as the ameloblastic component, the duct-like structures, the cribriform architecture, the whorled cell condensations like morulae, and the dentinoid component found in two-thirds of these lesions. This tumor has an aggressive biological behavior, with local infiltration and a recurrence of 45.5%–70%. It is commonly diagnosed when destruction of the cortical layers is found; it is generally asymptomatic; however, it can cause pain and paresthesia in some cases. Radiographically, it can be observed as a radiolucent lesion that may have a radiopaque component inside. This tumor has very few reports, about 40 until its publication as a new entity; however, it has not yet been determined whether it is a variant of ameloblastoma or a different tumor [9].

The metastatic ameloblastoma has remained in this group since the 2017 classification in which this lesion was removed from the group of malignant tumors described in 2005; however, its high mortality rate (30%) and its ability to metastasize make it controversial to still consider it a benign odontogenic tumor [9], which means that in our opinion, this entity can be considered a malignant odontogenic lesion.

### 3.4. Location and Clinical Characteristics

It often presents as a slow, asymptomatic growth that causes expansion or perforation of the cortical bone [7]; however, it can cause pain if hemorrhage is present at the adjacent soft tissues [20]. The solid variant is the most common, covering 75% of all cases, with 80% appearing in the posterior region of the jaw. The second most common type of ameloblastoma is the unicystic, constituting approximately 20% of all cases, with a great predominance for the jaw. The third type of ameloblastoma is peripheral with less than 5% of the reported cases, generally occurring at the gingival mucosa in the posterior mandibular region, occasionally presenting minimal bone erosion [18].

Approximately 80% of cases are found at the mandible, generally at the region of the third molar, while 20% are located at the posterior region of the maxilla, except for the desmoplastic subtype, which is located more frequently at the anterior and premolar region of the mandible or maxilla [20].

The presence of this tumor has been documented in syndromes, the most important being the Gorlin syndrome, in which the predilection are females and at the maxilla, differing from what is usual. Other syndromes in which the presence of ameloblastomas has been described are the epidermal nevus syndrome, the Gardner syndrome, the Simpson–Golabi–Behmel syndrome, and the Williams syndrome [7].

### 3.5. Imaging Characteristics

Imaging studies are of vital importance for the diagnosis and treatment plan of this type of lesions [22], which can be visualized in an orthopantomography or CT in which a radiolucent/hypodense osteolytic image can be observed; however, it must be taken into account that the desmoplastic variant can be observed as a mixed radiolucent area with diffuse radiopacities, similar to a fibro-osseous lesion [23]. It may be associated with an unerupted dental organ [24]. It can be observed as a unicystic or multicystic image, which generally adopts two patterns known as “soap bubbles” or “honeycombs” corresponding to large or small lattices, respectively [18, 24]; however, it is worth mentioning that the bone septa formed inside the lesion does not necessarily indicate a type of multicystic ameloblastoma [22]. The pathology is usually 2–8 mm larger than what is seen on an X-ray [25]. Resorption of adjacent tooth roots has also been observed [18, 24].

The differential diagnoses of ameloblastoma are varied and include pathologies of different natures, such as keratocyst [22], odontogenic myxoma, central giant cell tumor [23], dentigerous cyst [26], osteomyelitis, cystic fibrous dysplasia, ossifying fibroma, dentigerous cyst, multiple myeloma and even sarcomas, adenocarcinomas, and squamous cell carcinomas when they involve adjacent soft tissue [19]. The definitive diagnosis must be obtained by a histopathological study [27].

### 3.6. Genetic Mutations

In ameloblastomas, BRAF is the most frequently mutated gene, having a mutation rate of 63% [28, 29]. Some studies have found that BRAF mutations frequently coexist with RAS and FGFR2 gene mutations in the MAPK pathway. In addition, mutations in the EGFR genes, the homolog of the KRAS viral oncogene, and NRAS are also present in ameloblastomas. Activation of the MAPK pathway leads to cell proliferation, cell survival, inhibition of apoptosis, and other processes that promote tumor development [29].

The SHH pathway plays an important role, such as dental development. In this pathway, the SHH ligand binds to PTCH1, which promotes the inhibition of the SMO protein, and SMO signaling activates GLI transcription factors, leading to induction of genes involved in cell differentiation or proliferation [30, 31]. BRAF mutation occurs most frequently in the mandible [32, 33], while SMO mutation occurs mainly in the maxilla. The presence of SMO and BRAF mutations may be mutually exclusive [33].

### 3.7. Treatment

A radical surgical treatment is usually considered when recurrence appears after conservative treatment of a solid ameloblastoma. However, its treatment is quite controversial due to the opposite currents of solution. Radical treatment consists of an en bloc resection with safety margins to avoid recurrences; this type of treatment entails significant morbidity for the patient; however, the current trend is a conservative treatment such as enucleation/curettage/peripheral ostectomy for the unicystic type [10, 18]. This is not the case for the solid/multicystic type, because this lesion can extend towards the medullary bone up to 8 mm beyond the radiographic margin, so it is recommended to maintain a margin of at least 1 cm due to its high recurrence rate when curettage is made, showing a 90% recurrence rate in a 2–3 years follow-up [18]. The follicular and plexiform types can overpass the edge of the tumor with 0.5–0.75 cm; the granular ameloblastoma presents a greater invasion of 0.75 cm; however, we must consider that ameloblastomas can have a histological predominance but may still have different subtypes [10].

Unicystic ameloblastomas react better to conservative treatment [18]; this due to less medullary invasion, showing an infiltration of approximately 0.25 cm from the tumor edge, with absence of pathology at a distance of 0.5 cm [10]. In these pathologies, decompression and subsequent marginal resection can be used with adjuvant therapies (Carnoy's solution, cryotherapy, and 5-fluorouracil), having a recurrence ranging from 10% to 20%, except when a mural invasion is found; in this situation, recurrences will go up to 60% as reported [18, 34]; this is why its reclassification as a conventional type was proposed for the fourth edition of the *WHO Classification of Head and Neck Tumours*; however, it was not reclassified and stood as unicystic ameloblastomas [35]. Another criterion that can be considered for resection is to debride the anatomical plane adjacent to the one that is clinically and radiographically affected [18, 34].

In cases where the ameloblastoma becomes unresectable due to its anatomical position, especially in lesions that extend to the base of the skull or within it, proton therapy could be used [18] with a dose similar to those used for carcinomas, which means a range from 66GY to 74.4GY taking into consideration the size of the pathology [25].

Peripheral ameloblastoma generally resolves with surgical excision of the lesion [18].

Currently, computer-guided surgery is a trend to improve the prognosis and results through reproducible digital planning through the manufacture of surgical guides and transsurgical navigation; some of the other advantages are shorter surgical time, lower final cost, and greater precision [18].

Another treatment trend is to focus on the genetic characteristics of this lesion, emphasizing on the use of drugs for molecular therapy, inhibiting the functions of the BRAF and MEK mutations. The drugs developed until now are vemurafenib and dabrafenib, both of which can inhibit the mutated BRAF gene; trametinib inhibits the mutated MEK gene; ponatinib and regorafenib inhibit the mutated FGFR2 genes; this therapy needs more studies; however, it is promising due to the possible reduction of radical surgeries and the morbidity that they prevent [8, 21].

### 3.8. Malignant Transformation

An ameloblastic carcinoma can be originated as a malignant transformation of a preexisting ameloblastoma; however, this carcinoma generally develops as a new entity, not depending on a previous pathology, constituting 30% of the malignant odontogenic tumors; this ameloblastoma transformation is rare but possible and it usually originates from a long-standing ameloblastoma, which has not been treated or has had repeated postsurgical recurrences [32, 35].

The mechanism of oncogenic transformation has been discussed, and different possibilities have been hypothesized, among which the postsurgical inflammation of a recurrent lesion is mentioned; molecular and genetic factors are not yet fully described but have been attributed to transcriptional silencing through methylation of the p16 gene promoter [36]; the deregulation of multiple genes associated with mitogen-activated protein kinase, Sonic hedgehog, and WNT/b-catenin signaling pathways [32] and the important role of the tumor microenvironment play for its development; when a tumor grows rapidly, there is local ischemia and hypoxia; it is here that the hypoxia-inducible transcription factor 1*α* (HIF-1*α*) can work as a molecular sensor of oxygen tension that helps cells tumors to adapt to low oxygen levels; this factor can induce the epithelial–mesenchymal transition (EMT) that has been found in hepatocellular carcinomas and is involved in tumor invasion and metastasis. Another transcription factor that can induce EMT and that has been implicated in the malignant transformation of ameloblastoma is the zinc finger E-box binding homeobox 1 (ZEB1). It is important to know that EMT constitutes a phenotypic change in embryogenic development and tissue remodeling. When this occurs, cells lose intercellular adhesion, morphology is altered, and mobility increases. Malignant transformation of ameloblastoma has been attributed to hypoxia-induced HIF-1*α* and ZEB1 expression via TGF-*β*-dependent EMT. This means that HIF-1*α* and ZEB1 could be used as biomarkers to predict malignant transformation [37].

Transformation to ameloblastic carcinoma is not the only malignancy that an ameloblastoma can undergo; the transformation of desmoplastic ameloblastoma to squamous cell carcinoma has been reported; however, the mechanism of oncogenesis is unknown, but p53 overexpression has been observed in the carcinoma suggesting a possible implication [38].

### 3.9. Follow-Up

Tomographic and radiographic follow-ups should be done every 6 months for the first 5 years; after that, the follow-up should be changed to once a year for the next 5 years; after the first decade of follow-up, radiographic controls can be done every 2–3 years for the next 15 years, completing a total of 25-year follow-up [15].

## 4. Discussion

The treatment for ameloblastoma can be controversial and debatable; when talking about a conservative treatment, we refer to decompression, marsupialization, curettage, and/or enucleation that can be associated with adjuvant therapies such as cryotherapy, Carnoy's solution, and 5-fluorouracil, causing lower morbidity with better esthetic and functional results for patients when compared to radical treatments such as marginal or segmental resection of the affected site with safety margins; however, recurrence with conservative treatment is controversial [39]. In the case we present, decompression was considered the treatment option due to the diagnosis of unicystic ameloblastoma with which the patient arrived at our clinic; however, when we performed the second biopsy during the installation of the decompression devices to confirm the diagnosis, the histopathological result was different with a diagnosis of a follicular type of multicystic ameloblastoma; it was then that radical treatment such as marginal resection was considered; however, upon observing a reduction in the diameter of the pathology at the first month of treatment, the decompression devices were left in place to continue the initial protocol treatment.

Other authors have also mentioned that the treatment should be decided depending on several factors such as tumor type, anatomical location, extent of the lesion, histological and imaging characteristics, patients age, and his commitment to adhere to the treatment as mentioned by Hasegawa et al. [15]; in addition, we should consider that a conservative treatment, being less invasive, will help maintain and improve the patient's quality of life; the surgical time will also be reduced, but we should keep in mind that there will be a greater risk of recurrence (41%–50%) [12, 40] and a possible need to make other surgical interventions; meanwhile, a radical treatment minimizes recurrence (8%–12%) [12, 40] but with a greater risk of postsurgical complications, fewer prosthetic rehabilitation options, and the need of more surgical reconstructive interventions [12]. Additionally, we must take into consideration the consequences of aggressive treatments, such as masticatory dysfunction, mutilation, deformity, and abnormal mandibular movements that considerably affect the patient's quality of life [15].

In a retrospective study conducted by Boffano et al. [40], they showed that enucleation was the conservative treatment with the lowest recurrence rate (only 3% below marginal resection), a situation that, according to the authors, was because the success of the treatment will depend on the adequate case selection. Nevertheless, clinical and radiographic follow-ups in these patients are important to diagnose recurrences early, generally solved with radical treatments; however, a comprehensive evaluation of the patient is important to assess a possible conservative management.

Effiom et al. mention that the clinical–histological relationship of the tumor will determine the aggressiveness and dictate the treatment, without failing to consider the opinion of the patient and his general health status; however, they mention that if recurrence is the decision-making factor, then radical treatment should be the first option indistinguishable from the patient's age group, taking as an exception only patients with systemic impediments to said intervention [32]. On the contrary, Hendra et al. mention that in pediatric patients, conservative treatment with decompression or enucleation plus Carnoy's solution will always be preferable, indistinguishable from the type of ameloblastoma, avoiding facial growth alterations and psychological, functional, or esthetic disorders [12]. Other authors have also performed conservative treatments for multicystic ameloblastomas, such as Huang et al., who conducted a study in which they treated unicystic and solid/multicystic ameloblastomas in pediatric patients conservatively, with enucleation in combination with peripheral ostectomy, with decompression with a subsequent enucleation making a peripheral ostectomy at the end, or an aggressive treatment with segmental resection and iliac crest graft application; it is described that decompression should be considered the first treatment option if with the incisional biopsy the tumor has a cystic component in the histopathological study, whether it is a unicystic or multicystic ameloblastoma [11]. In our case, a cystic pattern was observed microscopically, and despite not being a pediatric patient as suggested by Huang, a conservative treatment was performed with good functional and esthetic results.

Neagu et al. suggest that conservative treatment such as enucleation, curettage, or marsupialization with adjuvants such as cryotherapy or Carnoy's solution should only be used to treat unicystic or multicystic lesions if they are small in size, while radical treatment is suggested only for advanced solid or unicystic ameloblastomas, all to minimize the risk of recurrence and therefore reduce the number of complementary surgeries; they also propose reconstructive treatment and new digital techniques as an important tool in case of radical treatment [39]. On the contrary, Hasegawa et al. recommend conservative treatment whenever possible. In their study, they performed marsupialization with enucleation, enucleation with curettage, and enucleation alone in solid/multicystic ameloblastomas, with the last treatment option being the one with the most recurrences; however, they propose to be conservative rather than aggressive, to optimize the patient's quality of life, with an adequate monitoring that allows timely diagnosis and treatment in case a new intervention is necessary, a position in which we agree, as long as the patient is well informed of the possibility of future surgical interventions in the case of recurrence, generally 2–5 years after the initial treatment [15, 18] and its possible, but rare, malignant transformation reported in the literature [41].

While no complications were encountered in the case presented, as with any surgical procedure, there are potential risks when applying this technique. Some of the most probable complications include infection of the tumor due to bacterial contamination through the drains; this is treatable with antibiotic therapy and irrigation with an antimicrobial solution until the infection resolves. Other potential complications include nerve injury or damage to adjacent anatomical structures during the ostectomy, which can be minimized with an adequate depth planification. Mandibular fracture is another potential complication; to minimize the risk, adequate planning and postsurgical dietary measures are important.

Recurrence is the main limitation of a conservative management of a multicystic ameloblastoma; however, in our opinion, this is an acceptable risk to prolong the patient's quality of life before resorting to radical treatment. A close follow-up is crucial for early detection of recurrence, allowing less aggressive interventions. Successful management relies on patient adherence to treatment protocols and follow-up, making it unsuitable for all patients. Therefore, treatment individualization is crucial.

Our article is limited to a single case; despite this, it would be of great clinical–surgical interest to conduct studies with biomarkers with a larger number of patients to understand the biological behavior before and after decompression with enucleation. It is also essential to carry out studies with long-term follow-ups to determine the association between conservative treatment and increased aggressiveness or malignancy.

## 5. Conclusions

Conservative treatment for multicystic ameloblastoma is a controversial and complex issue due to many factors that intervene in the therapeutic decision; in our opinion, a risk–benefit analysis for the patient should always be evaluated, trying to be as conservative as possible, even if it means a potential future reintervention, which could only result in a new conservative treatment, without the need to perform a mutilating treatment that could affect the patient's quality of life.

We suggest that the primary treatment for solid ameloblastoma with a cystic pattern should be decompression followed by enucleation with peripheral ostectomy, always with a close short- and long-term follow-up. Patients should always be clearly informed of all the risks and complications arising from conservative and radical treatment.

## Figures and Tables

**Table 1:** Classification of ameloblastoma.

**Unicystic**	**Solid/multicystic**	**Adenoid**	**Peripheral**	**Metastasizing**
Luminal	Follicular			
Intraluminal	Plexiform			
Mural	Desmoplastic			
	Acanthomatous			
	Granular cells			
	Basal cells			

*Note:* Based on the *WHO Classification of Head and Neck Tumours*. Fifth Edition (2022) [9].

## Data Availability

The authors have nothing to report.
